# Bacteriophage-based decontamination to control environmental colonization by *Staphylococcus capitis* in neonatal intensive care units: An *in vitro* proof-of-concept

**DOI:** 10.3389/fcimb.2022.1060825

**Published:** 2022-11-16

**Authors:** Marie Chavignon, Camille Kolenda, Mathieu Medina, Mélanie Bonhomme, Leslie Blazere, Tiphaine Legendre, Anne Tristan, Frédéric Laurent, Marine Butin

**Affiliations:** ^1^ Centre International de Recherche en Infectiologie (CIRI), Team « Pathogénie des Staphylocoques », Inserm, U1111, Centre national de la recherche scientifique (CNRS) UMR5308, Ecole normale supérieure (ENS) Lyon, Université Claude Bernard Lyon 1, Lyon, France; ^2^ Institut des Agents Infectieux, Centre National de Référence des Staphylocoques, Hospices Civils de Lyon, Lyon, France; ^3^ Service de Néonatologie et Réanimation Néonatale, Hôpital Femme Mère Enfant, Hospices Civils de Lyon, Lyon, France

**Keywords:** *staphylococcus capitis* NRCS-A, neonatal intensive care units (NICU), bacteriophages, biofilm, disinfection

## Abstract

**Introduction:**

In neonatal intensive care units (NICUs), the standard chemical-based disinfection procedures do not allow a complete eradication of pathogens from environmental surfaces. In particular, the clone *Staphylococcus capitis* NRCS-A, a significant pathogen in neonates, was shown to colonize neonatal incubators. The aim of this study was to evaluate the *in vitro* effect of a bacteriophage cocktail on NRCS-A eradication.

**Methods:**

Three bacteriophages were isolated, genetically characterized and assessed for their host range using a collection of representative clinical strains (n=31) belonging to the clone NRCS-A. The efficacy of a cocktail including these three bacteriophages to eradicate the reference strain *S. capitis* NRCS-A CR01 was determined in comparison or in combination with the chemical disinfectant Surfanios Premium on either dry inoculum or biofilm-embedded bacteria. The emergence of bacterial resistance against the bacteriophages alone or in cocktail was evaluated by growth kinetics.

**Results:**

The three bacteriophages belonged to two families and genera, namely *Herelleviridae/Kayvirus* for V1SC01 and V1SC04 and *Rountreeviridae/Andhravirus* for V1SC05. They were active against 17, 25 and 16 of the 31 tested strains respectively. Bacteriophage cocktails decreased the bacterial inoculum of both dry spots and biofilms, with a dose dependent effect. The sequential treatment with bacteriophages then Surfanios Premium did not show enhanced efficacy. No bacterial resistance was observed when using the bacteriophage cocktail.

**Discussion:**

This study established a proof-of-concept for the use of bacteriophages to fight against *S. capitis* NRCS-A. Further investigations are needed using a larger bacterial collection and in real-life conditions before being able to use such technology in NICUs

## Introduction

Nosocomial infections in hospitalized patients are a well-known cause of mortality and morbidity all over the world (Sikora and Zahra, 2022). The prevalence of such infections is high, representing 6.5% of the patients in the European Economic Area and 3.2% patients in the United States (Magill et al., 2018; Suetens et al., 2018). Multidrug resistant bacteria from the hospital environment are frequently involved in nosocomial infections (Suleyman et al., 2018). Among the various hospital units, neonatal intensive care units (NICUs) are at high risk of nosocomial infections since preterm neonates are highly vulnerable given their global immaturity (Collins et al., 2018). Despite the strict hygiene measures implemented in NICUs, several studies have incriminated numerous pathogens in those infections including staphylococci, enterobacteria or enterococci originating from the environmental surfaces and notably from the incubators where preterm newborns are housed (Golan et al., 2005; Lin et al., 2017; Carter et al., 2018; Butin et al., 2019; Cadot et al., 2019; Ory et al., 2019; Chavignon et al., 2021).

The clone *Staphylococcus capitis* NRCS-A is one of these pathogens. Its high prevalence in sepsis among low-birth weight preterm neonates in NICUs worldwide, its multidrug resistant profile and its high adaptation to the NICU environment have been reported (Butin et al., 2017; Wirth et al., 2020). This specialization has been related to its resistance pattern to antibiotics commonly used in NICUs including vancomycin or aminoglycosides (Rasigade et al., 2012; Wirth et al., 2020). Moreover, previous studies have highlighted the ability of NRCS-A to colonize inert surfaces, notably neonatal incubators, and to persist despite the usual chemical disinfection procedures fostering its continuous spread and dissemination after its initial introduction in a given NICU (Carter et al., 2018; Butin et al., 2019; Chavignon et al., 2021). Finally, the ability of *S. capitis* strains isolated from neonates or from the NICU environment to form biofilm has been highlighted and might explain, at least for a part, the ability of the strains to colonize abiotic surfaces (Cui et al., 2013; Carter et al., 2018; Qu et al., 2020). In this context, there is a need to develop alternative disinfection approaches to reduce the risk of environmental persistence of *S. capitis* and inter-patient transmission. One innovative approach could be the setup of bacteriophage-based decontamination. Bacteriophages are ubiquitous viruses able to specifically infect prokaryotic bacterial cells with a total innocuity for eucaryotic cells and so for humans (D’Accolti et al., 2021). Thus, lytic virulent bacteriophages present a bactericidal activity with a high target specificity at the species or infra-species level and constitute useful tools for biological control. The use of bacteriophages has already been considered as an alternative or a complementary method of decontamination in different contexts (D’Accolti et al., 2021). In food-processing industries, the use of bacteriophages as additive to control the contamination of food production by pathogenic bacteria has been already approved by the Food and Drug Administration (https://www.cfsanappsexternal.fda.gov/scripts/fdcc/?set=GRASNotices&sort=GRN_No&order=DESC&startrow=1&type=basic&search=phages, retrieved 9 August 2022). Bacteriophages have also been considered in decontamination of inanimate surfaces in the hospital environment. For instance, the use of an aerosolized bacteriophage suspension after the standard cleaning procedure (based on sodium hypochlorite 0.06% and alcohol 75%) in an intensive care unit in Taiwan has been reported to be able to decrease the rate of nosocomial infections caused by carbapenem-resistant *Acinetobacter baumannii* (Ho et al., 2016). In another study, D’Accolti et al. demonstrated the feasibility and effectiveness of hospital bathrooms nebulization with bacteriophages in addition to a probiotic-based sanitation to fight against *Staphylococcus* spp. contamination and nosocomial infections. Interestingly, daily nebulization of bacteriophages allowed a significant and rapid decrease of *Staphylococcus* on the surfaces that was up to 97% more effective than probiotic-based sanitation alone (D’Accolti et al., 2019).

Due to the difficulty to eradicate the clone *S. capitis* NRCS-A after its initial introduction in a NICU and to face its persistence, an *in vitro* preliminary study was conducted to determine if a bacteriophage-based decontamination could be an efficient approach. The aim of the present study was thus i) to isolate lytic bacteriophages presenting a bactericidal activity on clinical strains belonging to the clone *S. capitis* NRCS-A, ii) to explore the emergence of resistance against those bacteriophages, and finally iii) to evaluate the ability of those bacteriophages to impact biofilms or planktonic adhered bacteria in comparison with the usual disinfection molecule.

## Materials and methods

### Bacterial strains

The *Staphylococcus capitis* strains used in this study were obtained from the collection of the French National Reference Centre for Staphylococci (Lyon, France). These strains were conserved in cryotubes containing glycerol at -20°C. They were grown on Columbia agar + 5% sheep blood (BioMérieux, Marcy l’Etoile, France) for 24 hours at 37°C. A collection of 31 strains was included in this study to isolate, produce and assess the host range of anti-*S. capitis* bacteriophages. These strains were selected to reflect the phylogenetic diversity of the *S. capitis* NRCS-A clone composed of three subgroups successively appeared: Proto-outbreak I, Proto-outbreak II and the most recent and specialized in NICU infections Outbreak (**Table 1**) (Wirth et al., 2020). The reference strain of the clone *S. capitis* NRCS-A, called CR01 (Lemriss et al., 2014) has been used to evaluate the effect of bacteriophages on dry spots and biofilms.

**Table 1:** Host range characterization of five anti-Staphylococcus capitis bacteriophages.

Bacterial strains			Bacteriophages EOP		
			*Kayvirus*		*Andhravirus*
Strain number	Strain in Wirth et al., 2020	Clade in Wirth et al., 2020	V1SC01	V1SC04	V1SC05
P2 SC17	AD78	Outbreak	7E-02	3E-01	0E+00
P2 SC18	AE19	Outbreak	0E+00	3E-01	0E+00
P2 SC19	AE20	Outbreak	0E+00	1E-01	0E+00
P2 SC20	AE21	Outbreak	0E+00	3E-01	0E+00
P2 SC21	AE22	Outbreak	2E-04	2E-01	5E+02
P2 SC22	AE23	Outbreak	3E-04	3E-01	1E+03
P2 SC23	AV75	Outbreak	7E-06	1E-01	0E+00
P2SC04	BI33	Outbreak	0E+00	2E+01	0E+00
P2SC09	CR01^a^	Outbreak	1E+00	1E+01	1E+01
P2SC10	AL07	Outbreak	2E+00	2E+01	0E+00
P2SC16	AD75	Outbreak	3E-01	9E-01	4E+02
P2SC24	AW16	Outbreak	2E+00	2E+01	0E+00
P2SC25	AW17	Outbreak	3E+00	2E+01	0E+00
P2SC33	AV74	Outbreak	0E+00	3E-01	6E+02
P2SC34	AW19	Outbreak	0E+00	1E+00	3E-02
P2SC35	AW77	Outbreak	7E-01	1E+00	4E-02
P2SC36	BA22	Outbreak	1E-01	4E-02	1E+02
P2SC37	BC08	Outbreak	3E-03	8E-01	3E+02
P2SC01	BI76	Proto-outbreak II	0E+00	0E+00	0E+00
P2SC06	AW20	Proto-outbreak II	0E+00	0E+00	1E+00
P2SC38	BC76	Proto-outbreak II	0E+00	0E+00	0E+00
P2SC39	BD01	Proto-outbreak II	0E+00	0E+00	5E+00
P2SC40	BD06	Proto-outbreak II	5E-01	0E+00	0E+00
P2SC41	BI77	Proto-outbreak II	0E+00	4E-05	0E+00
P2SC02	BD61	Proto-outbreak I	1E+00	1E+00	1E+00
P2SC03	BA08	Proto-outbreak I	5E-01	1E+00	0E+00
P2SC42	AK81	Proto-outbreak I	1E+00	1E+00	3E+02
P2SC43	BD62	Proto-outbreak I	9E-01	8E-01	8E+01
P2SC44	BG77	Proto-outbreak I	5E-01	6E-01	9E+01
P2SC45	AL04	Proto-outbreak I	1E+00	9E-01	1E+03
P2SC46	BA10	Proto-outbreak I	5E-01	1E-01	0E+00

^a^Strain used for testing bacteriophage cocktail and Surfanios Pemium for eradication of dry spots and biofilms and for evaluating the development of bacterial resistance againstbacteriophages. Mean EOP scores calculated from 3 biological replicates are indicated (red: EOP ≤ 10-3 blue: 10-3 < EOP ≤ 10-1 red: EOP > 10-1).

### Bacteriophages isolation and production

Three bacteriophages were isolated for the purpose of this study from three different wastewater samples in Lyon, France in 2020. Briefly, 5 mL of filtered water sample was incubated during 24 h at 37°C under agitation (180 rpm) with 500 µL of Tryptic Soy Broth (TSB) 10X (BD, Franklin Lakes, NJ, USA) and 10 µL of an overnight culture of the strain P2SC02, chosen for the intensity of the lysis produced by the bacteriophages when applied to it. The culture supernatant was filtered using a 0.22-µm syringe filter and diluted in double layer agars pouring a mix of 100 µL of this supernatant, 250 µL of P2SC02 culture and 5 mL of medium containing 0.75% of agar (called TSB soft-agar) over a Tryptic Soy Agar plate (BioMérieux, Marcy l’Etoile, France). Individual plaque forming units (PFU) were further purified by 5 rounds of serial passages and where required propagated in liquid medium. Strain P2SC16 was then used for bacteriophage production as amplification yields were higher with this strain. Bacteria were cultured in 50 mL of TSB in exponential phase then inoculated with bacteriophages to obtain a Multiplicity of Infection (MOI) of 10^-3^. Obtained bacteriophage lysates were filtered and stored at 4°C.

### Bacteriophage genome sequencing and bio-informatic analysis

After isolation, a volume of 6 mL of each bacteriophage was centrifuged at 14,000 g for 5 h and pellets were re-suspended in 50 µL of NaCl 0.9%. Enzymatic treatment with 100 mU of benzonase^®^ nuclease (Merk, Darmstadt, Germany) at 37°C overnight was then used to degrade extracellular bacterial DNA, followed by benzonase heat-inactivation at 95°C for 30 min, a treatment with 4 µg of proteinase K (Merk, Darmstadt, Germany), and a proteinase K heat-inactivation at 95°C for 30 min. Bacteriophage DNA was extracted using the DNA Extractor^®^ WB kit (Fujifilm, Osaka, Japan) and sequenced on an Illumina NextSeq 500 instrument using a 150-bp paired-end protocol. Reads were trimmed (cutadapt, v3.4; trimmomatic, v0.39) and the reads mapping against the genome of the P2SC16 strain used for the amplification of bacteriophages (bowtie2, v2.3.4.1; samtools, v1.15) were removed. The remaining reads were assembled (SPAdes, v3.13.0) and scaffolds smaller than 100 bp were removed. Taxonomic assignation was performed using kraken 1.1.1 with the minikraken database. Genomes were annotated using PATRIC (v3.6.12) with parameters for bacteriophage annotation. Finally, Abricate (v0.8.13) was used for resistance and virulence gene detection using all the databases available. The lytic nature of bacteriophages was assessed using the Phage AI repository (https://app.phage.ai/phages/). Bacteriophage genomes were deposited on the NCBI GenBank under the accession numbers OP323059, OP297178 and OP297179 for vB_ScaM-V1SC01, vB_ScaM-V1SC04, and vB_ScaM-V1SC05, respectively, which are named V1SC01, V1SC04, and V1SC05 in this study for simplification.

### Host range assessment

Host range of each bacteriophage was assessed using the spot test assay on the panel of 31 *S. capitis* strains, selected as explained above (**Table 1**). Briefly, 5 µL of serial ten-fold dilutions of bacteriophages were spotted on an agar plate prepared extemporaneously by mixing 30 mL of TSB soft agar and 500 µL of *S. capitis* overnight culture in TSB broth. After overnight incubation at 37°C, PFU were enumerated. The efficiency of plating ratio (EOP) was calculated dividing the bacteriophage titer obtained on a test strain by the titer of the same bacteriophage suspension on the reference production strain P2SC16. A bacteriophage was considered active if EOP was ≥ 0.001 (Green et al., 2017). Experiments were performed in biological triplicates and mean EOP scores were calculated.

### Evaluation of bacterial resistance emergence during bacteriophage treatment

Growth kinetics were performed to determine if bacterial resistance occurred following the treatment with one bacteriophage alone compared to cocktails of the three bacteriophages. Briefly, CR01 was incubated in TSB until the exponential growth phase and then diluted in TSB to reach a concentration of 1.10^7^ CFU/mL. A 96 well microplate (Thermo Fisher Scientific, Waltham, MA, USA) was then inoculated with 1.10^6^ CFU of bacteria and either 1.10^6^ PFU, 1.10^7^ PFU or 1.10^8^ PFU of each bacteriophage alone or in cocktail (to reach a MOI of respectively 1, 10 or 100) in a final TSB volume of 200 µL. Bacterial growth alone without bacteriophage addition and absence of bacterial contamination in bacteriophage suspensions and in TSB were also controlled in the microplate. The outlines of the microplate were filled with water to prevent evaporation then the microplate was incubated 24 h in a microplate reader Tecan infinite^®^ 200 PRO (Tecan, Männedorf, Swiss) under agitation at 37°C with OD_600 nm_ (optical density) measurement every 30 min. Emergence of bacterial resistance was considered when the OD_600 nm_ exceeded the threshold of 0.01 (after TSB control media OD_600 nm_ subtraction) that corresponded to the threshold of bacterial growth. The experiments were performed three times in technical triplicates and both bacteriophage titres and bacterial inoculum were verified each time.

### Activity of a bacteriophage cocktail on dry spots and pre-formed biofilm of *S. capitis* NRCS-A

The effect of a bacteriophage cocktail was tested on the reference strain CR01 in comparison with the disinfectant Surfanios Premium (SP) (composed of N-(3-aminopropyl)-N-dodécylpropane-1,3-diamine and chlorure de didécyldiméthylammonium) (ANIOS, Lezennes, France). Both treatments were tested on dry spots and on pre-formed biofilms using a previously published method with some modifications (Stachler et al., 2021). For bacteriophage cocktails preparation, the three bacteriophages were associated by diluting each of them in equal measure in either TSB or Phosphate Buffered Saline (PBS) (Thermo Fisher Scientific, Waltham, MA, USA) to obtain cocktails at three different final concentrations (3.10^4^ PFU/mL, 3.10^6^ PFU/mL or 3.10^8^ PFU/mL). Bacteria were exposed to the bacteriophage cocktail for 6 h in wet chamber at 37°C. The chemical treatment consisted in exposition to SP at 0.25% in water at room temperature for 20 minutes, corresponding to the classical treatment used in NICUs for incubator disinfection (Butin et al., 2019). Untreated positive controls were treated with either TSB or PBS (controls for bacteriophage treatments) or water (control for SP treatment) alone.

Dry spots were obtained by inoculating 10 µL drops of a 1.10^7^ CFU/mL bacterial PBS suspension in a 96 well microplate (Greiner Bio-One, Cap Horn, France). The drops were dried at room temperature during 4.5 h under microbiological safety workstation. The dry spots treatment consisted of covering the adhered bacteria with 20 µL of bacteriophages cocktail, SP or control medium. Then, 100 µL of Dey Engley Neutralizing broth (Merk, Darmstadt, Germany) was applied during 5 min to inactivate the SP action. This same neutralising treatment was applied to dry spots exposed to bacteriophages to avoid experimental variations. Finally, the neutralising broth was discarded and the surviving bacteria were suspended in 100 µL of PBS by scraping and enumerated.

Biofilm was obtained by inoculating 100 µL of a 1.10^7^ CFU/mL bacterial suspension in TSB in a 96 well microplate that was incubated 24 h at 37°C. After incubation, the biofilm was first rinsed using the steam method as previously described (Tasse et al., 2018) before being exposed to 150 µL of bacteriophage cocktails or SP 0.25% as described above. After incubation, the supernatant was carefully removed and 200 µL of Dey Engley Neutralizing broth was applied for 5 minutes. The biofilm was then rinsed with the steam method, suspended in 200 µL of PBS by scraping and enumerated.

In addition, a sequential treatment with a bacteriophage cocktail at the highest bacteriophages concentration (3.10^8^ PFU/mL) followed by a SP treatment was also evaluated on bacterial biofilm. For this purpose, after the step of bacteriophage treatment as described above, an additional steam rinse was performed and then SP was applied followed by inactivation as previously described.

Log10 reduction values (LRV) for each treatment were calculated as follows: Log10(N/N0) with N corresponding to the number of bacteria in the treated well and N0 corresponding to the number of bacteria in the untreated control well. The experiments were performed three times in triplicate for the simple treatments and three times in sextuplicate for the sequential treatment. Bacteriophage cocktails titres were checked each time.

### Graphic representation and statistical analyses

The graphics were drawn on GraphPad Prism 8 (La Jolla, CA, USA) using the scatter dot plot representation with the mean and the standard deviation displayed for each condition. Statistical analyses were performed in GraphPad Prism 8 on the LRV values. Because of the small sample size in the different experiments (experiments performed three times in triplicate or in sextuplicate), the Mann-Whitney nonparametric test was performed, with an α risk of 0.05.

## Results

### Bacteriophages characteristics and host range against *S. capitis* NRCSA

The three bacteriophages belonged to two different families and genera, namely *Herelleviridae/Kayvirus* for V1SC01 and V1SC04 or *Rountreeviridae/Andhravirus* for V1SC05. VISC04 had the widest host range as it was active against 25 out of the 31 tested strains while V1SC01 and V1SC05 were active against 17 and 16 of the strains respectively (**Table 1**). Of note, activities of bacteriophages were complementary: 28 out of the 31 strains were susceptible to at least one bacteriophage. However, bacteriophage activity depended on the NRCS-A subgroup: all strains of the Outbreak and Proto-outbreak I groups were susceptible to 1 to 3 bacteriophages, while 3 out of the 6 Proto-outbreak II strains were resistant to all bacteriophages.

### Emergence of resistances against bacteriophages

After 24 h of growth in presence of bacteriophages, growth curves indicated that CR01 did not develop resistance against the bacteriophage V1SC01 while resistance to V1SC04 at MOI 1 and MOI 100 and to V1SC05 at MOI 1, MOI 10 and MOI 100 were detected (**Table 2**). The emergence of these resistances revealed by bacterial regrowth always occurred in the late phase of the experiment (18 h to 24 h). Interestingly, no resistance was observed whatever the MOI when bacteriophage cocktail of the three bacteriophages was tested.

**Table 2:** Development of bacterial resistance against bacteriophages using bacteriophage alone or in cocktail.

Bacteriophages	Frequency of bacterial resistance (%)			Onset of resistance during the 24 h growth kinetic
	MOI 1	MOI 10	MOI 100	
V1SC01	0	0	0	–
V1SC04	11,11	0	11,11	22h30 to 23h30
V1SC05	11,11	100	100	18h15 to 23h30
Cocktail V1SC01-04-05	0	0	0	–

MOI, multiplicity of infection.

### Bacteriophage cocktail activity compared to Surfanios Premium on bacterial dry spots

**Figure 1:**
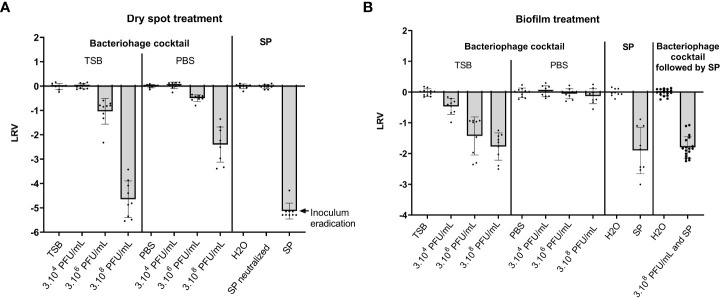
Effect of a bacteriophage cocktail and Surfanios Premium on dry spots and pre-formed biofilms of *S. capitis* NRCS-A. Results are presented as Log_10_ reduction values (LRV) calculated as Log10(N/N0) with N the remaining inoculum after treatment and N0 the remaining inoculum after treatment with diluent (TSB or PBS for treatment with bacteriophages and water for treatment with SP). The treatments consisted either of i) bacteriophage cocktails at three concentrations (3.10^4^ PFU/mL, 3.10^6^ PFU/mL or 3.10^8^ PFU/mL) in TSB or in PBS, ii) Surfanios Premium (SP) at 0.25% in water or iii) a sequential treatment with the bacteriophage cocktail at the highest concentration (3.10^8^ PFU/mL) in TSB followed by SP treatment. **(A)** Effect of the bacteriophage cocktails or SP individually on bacterial dry spots. **(B)** Effect of the bacteriophage cocktails or SP individually or sequentially on pre-formed biofilms.

While the SP 0.25% treatment was able to completely eradicate bacteria on the dry spots in the tested conditions, bacteriophage cocktail treatment significantly decreased the bacterial inoculum on dry spots both in TSB and in PBS at 3.10^6^ PFU/mL (1 LRV (p < 0.0001) and 0,5 LRV (p <0.0001) respectively) and at 3.10^8^ PFU/mL (4.7 LRV (p < 0.0001) and 2.4 LRV (p < 0.0001) respectively) but not at 3.10^4^ PFU/mL (**Figure 1A**).

### Bacteriophage cocktail activity compared to SP and sequential treatment on biofilm

Bacteriophage cocktail showed a bactericidal effect on biofilm only when incubated with TSB (**Figure 1B**). This bactericidal effect was significantly higher at 3.10^6^ PFU/mL and 3.10^8^ PFU/mL than at 3.10^4^ PFU/mL with 1.4 LRV and 1.8 LRV respectively versus 0.5 LRV (p = 0.0005 and p < 0.0001 respectively). The efficacy of SP 0.25% was not significantly different to that of bacteriophage cocktail at 3.10^8^ PFU/mL with 1.9 LRV (p = 0.6314).

The sequential treatment, consisting in a first exposition to bacteriophage cocktail at 3.10^8^ PFU/mL in TSB followed by an exposition to SP 0.25%, showed a 1.8 LRV (**Figure 1B**). This was not significantly different from the effect of either SP 0.25% alone or the bacteriophage cocktail alone at the highest concentration (p = 0.6959 and p = 0.6314 respectively).

## Discussion

The present study highlights the ability of a cocktail of three lytic bacteriophages to significantly reduce the bacterial inoculum of *S. capitis* NRCS-A on dry spots or in biofilm. Our data highlights that no emergence of resistance to the bacteriophage cocktail occurred during exposition of *S. capitis* NRCS-A. The sequential exposition to bacteriophages then to chemical disinfectant did not improve the final bactericidal effect observed.

Bacteriophages have to be considered in the perspective of environmental decontamination because they present advantages in comparison to chemical disinfectants. First, in contrast to bacteriophages, chemical products are generally toxic both for humans and the environment (Dhama et al., 2021). Thus, quaternary ammonium compounds (QACs), that are components of SP, have been previously shown to be harmful for frequently exposed healthcare workers by causing allergic contact dermatitis and asthma (Purohit et al., 2000; Suneja and Belsito, 2008). QACs exposition has also been shown to increase secretion of inflammatory cytokines, alter mitochondrial function, and disrupt cholesterol homeostasis in humans (Hrubec et al., 2021). All these features are of concerns, all the more when such disinfectants are used to clean incubators housing very low-birth-weight preterm neonates. Secondly, conventional disinfectants have a corrosive effect on abiotic surfaces (Song et al., 2019). Such damages may induce porosity on equipment surfaces which fosters biofilm formation and so might favor persistence of some pathogenic bacteria. In contrast, bacteriophages are usually presented as having no impact on human cells and so to be safe for human health in addition to be rapidly cleared from the human body, as previously shown in reports of bacteriophage therapy (Wright et al., 2009). Furthermore, due to their narrow spectrum of antibacterial activity, it seems that bacteriophages do not damage the beneficial microbiota and limit the selection of multidrug resistance (D’Accolti et al., 2021). However, it should be noted that despite these general statements, recent studies question the real neutrality of bacteriophages for eukaryotic organisms. Notably, due to their nature of protein-nucleic particle, bacteriophages were suspected to activate the immune system resulting in the production of anti-bacteriophages antibodies or the production of pro-inflammatory or anti-inflammatory cytokines (Dąbrowska et al., 2014; Yıldızlı et al., 2020; Podlacha et al., 2021). In addition, bacteriophages are able of transcytosis across confluent epithelial cell layers but also to interact with eukaryotic proteins, in particular protein misfolding and prion formation (Nguyen et al., 2017; Tetz and Tetz, 2017; Tetz and Tetz, 2018). Moreover, recent works suggested that bacteriophages could alter the normal microbiota, since Tetz et al. reported a high abundance of lytic *Lactococcus* bacteriophages associated with a reduction of *Lactococcus* in the microbiota of patients with Parkinson disease (Tetz and Tetz, 2018). For these reasons, in a perspective of using these bacteriophages for decontamination in a NICU, it would be necessary to carefully assess the safety of products containing bacteriophages.

The present study has been motivated by the need to explore innovative procedures for disinfection in NICU settings. The aim was to determine if bacteriophages could be used to fight against *S. capitis* NRCS-A environmental spread/persistence in NICUs. Our data documented a bactericidal activity of bacteriophages on both bacterial dry spots and biofilms. This action was dose dependant, as reported in previous studies (Zhang and Hu, 2013; Stachler et al., 2021). For instance, the bacterial decrease represented 4.7 LRV and 1.8 LRV respectively on dry spots and biofilms at 3.10^8^ PFU/mL in TSB while no bacterial loss on dry spots and only 0.47 LRV on biofilms was observed at 3.10^4^ PFU/mL. Our data demonstrated that the medium used for treatment impacted the efficacy of the bacteriophage cocktail on *S. capitis* including a better efficacy in TSB than in PBS, particularly on biofilm. This is consistent with the mechanism of action of bacteriophages that are known to better infect active bacteria (Azeredo and Sutherland, 2008; Harper et al., 2014). A rich medium, as TSB, could induce the re-growth of the dormant bacteria inside the biofilm and thus foster bacteriophages infection and replication inducing bacterial lysis. In addition, when bacteriophages greatly outnumber bacteria, they are able to kill it without intrabacterial replication, a phenomenon previously described as “lysis from without” (Abedon, 2011). This could explain the paradoxical efficacy of bacteriophages in PBS on dry spots at high bacteriophage concentration.

Despite all benefits of the use of bacteriophages, some points ought to be examined with particular attention. First, emergence of bacterial resistance against bacteriophages is frequent notably by avoiding bacteriophage adsorption or bacteriophage nucleic acid entry (based on alteration of bacteriophages nucleic acids or abortive infection systems) (Labrie et al., 2010; Oechslin, 2018). The emergence of bacterial resistance against bacteriophages was here prevented using a cocktail of three different bacteriophages, as already described in *in vitro* and *in vivo* studies (Yu et al., 2018; Yang et al., 2020). The choice of bacteriophages belonging to different genera (*Herelleviridae/Kayvirus* for V1SC01 and V1SC04 and *Rountreeviridae/Andhravirus* for V1SC05) may have been beneficial. Indeed, the Myoviridae including *Kayvirus* and the Podoviridae including *Andhravirus* are known to target different parts of wall teichoic acids in *S. aureus* (Moller et al., 2019). However, these are only hypotheses since in the present study, neither the mode of action of the bacteriophages nor their bacterial receptors have been investigated. It is likely that if these receptors are shared between two or three of these bacteriophages, it can increase the risk of bacterial resistance against the cocktail after a longer contact time than 24 h, as previously described (Oechslin, 2018). Moreover, our results demonstrate that the frequency of resistance increased for VISC05 used alone when the MOI increased. This result could be due to a faster selection of the mutant cells at higher bacteriophage concentration. This phenomenon has already been reported *in vivo* for *Salmonella* strains (Atterbury et al., 2007).

Given that the bacteriophage cocktail at the higher concentration showed a similar effect than SP on *S. capitis* biofilm without resistance acquisition, its use in a protocol of decontamination seems achievable. However, because we observed only an incomplete effect on biofilm of either SP or bacteriophage cocktails, we aimed to potentiate the bactericidal effect by testing a sequential treatment using SP exposition followed by bacteriophage cocktail exposition. Unfortunately no additive effect was observed. We hypothesize that the biofilm structure offered a protection for bacteria against those two treatments, even when they were sequentially administered, as already described for antibiotics or disinfectant molecules (Smith and Hunter, 2008; Schilcher and Horswill, 2020). In a previous study, Melo et al. reported that an anti-S*taphylococcus epidermidis* bacteriophage was ineffective against the bacteria in biofilm whereas it showed efficacy on released biofilm cells and persisters, a subset of the biofilm population, highlighting the protective effect of the biofilm matrix against bacteriophages activity. Indeed, the authors demonstrated by confocal microscopy that bacteriophages had access only to biofilm cells not embedded in N-acetyl-D-glucosamine residues (PNAG) (Melo et al., 2020). The biofilm tridimensional structure and architecture was also shown to protect against bacteriophages in other bacterial species, i.e. *Vibrio anguillarum* (Tan et al., 2015). Contrariwise, many bacteriophages can also exert damages on biofilms notably thanks to enzymes that target components of the biofilm matrix (Harper et al., 2014; D’Accolti et al., 2021). For instance, exopolysaccharide degradation by a bacteriophage depolymerase was identified as the first step in the disruption of an *Enterobacter agglomerans* biofilm (Hughes et al., 1998). In that regard, the use of bacteriophages polysaccharide depolymerases in association with disinfectants was demonstrated to be effective to remove an *Enterobacter* biofilm (Tait et al., 2002). The combination of lytic bacteriophage with anti-biofilm bacteriophage could lead to a synergistic effect on biofilm. It should be noted that no genes corresponding to polysaccharide depolymerases were identified in the genomes of the three bacteriophages used in the present study (data not shown).

According to our results showing the absence of total eradication of biofilm-embedded bacteria by a bacteriophage cocktail, the necessity of using a rich medium that can lead to growth of other environmental bacteria and knowing their narrow antibacterial spectrum, bacteriophages alone would not be sufficient to eradicate bacteria from NICU surfaces. Thus, if used, bacteriophages must be combined with other complementary methods in a perspective of environmental disinfection of NICUs. Previous studies have evaluated the efficacy of bacteriophages in association with usual disinfectants in situations of hospital bacterial outbreaks and have shown the effectiveness of these combinations (Roy et al., 1993; Zhang and Hu, 2013; Stachler et al., 2021). Of note, when bacteriophages and disinfectant molecules are tested in combination, it is required to determine the effect of each disinfectant on bacteriophages activity and vice versa to avoid antagonistic effects (Agún et al., 2018; Stachler et al., 2021). Based on our data which did not show a better efficacy of the sequential treatment with bacteriophages then a chemical disinfectant compared to each treatment alone, but also due to the potential adverse effects of chemical molecules, other alternatives have to be explored. Disinfection of the incubators using steam pulverization is also a safe, efficient and simple method that has already been implemented in several NICU settings (Braux et al., 2008; Gillespie et al., 2017; Ory et al., 2019). If it seems more efficient than chemical disinfection, it did not allow for a complete decontamination of incubators. In that context, it might be interesting to develop a disinfection protocol including both the steam pulverization method, which allows non-selective killing of bacteria, and a bacteriophage cocktail to specifically target the persistent NRCS-A strains. However, the order in which the bacteriophage cocktail and steam should be used remains to be defined. Such a protocol deserves to be tested in future studies. Besides, in a perspective of using bacteriophages in real-life decontamination, additional questions concerning production and safety of bacteriophages have to be addressed in futures works. In particular, this project would require to setup a method allowing a large production of the three bacteriophages in controlled conditions ensuring the absence of bacterial residues as toxins and pyrogens in the final product. Another critical point to control is the stability of the bacteriophages that must be maintained during their storage; previous studies have reported that this stability would be obtained if bacteriophages are stored at high concentration (1.10^9^ PFU/mL or more), or lyophilized in presence of sucrose or gelatin during months to years. (Pirnay et al., 2018; Manohar and Ramesh, 2019; Duyvejonck et al., 2021). Moreover, the safety of the treatment in the close environment of preterm neonates is another pivotal point but this will be easily performed since the product will be carefully rinsed after decontamination, to avoid the contact with neonates but also to avoid promoting the regrowth of other bacteria.

Finally, the present study presents several limitations. First, the tests confronting bacterial dry spots or biofilm to treatments were performed in a controlled laboratory setting using a single bacterial strain and a single type of material. Although CR01 is the reference strain for the NRCS-A clone, extended tests need to be performed to confirm the observed results on a larger collection of NRCS-A strains. Second, the comparison of the activity of bacteriophages and SP on dry spots has to be cautious given the differences in conditions during those two expositions. Indeed, during their treatment by bacteriophages, bacteria had grown in a rich medium (TSB) during 6 h versus water during 20 minutes for the SP treatment. Thus, the quantification of the effect using LRV is not fully comparable because the bacterial inoculum has evolved differently. Third, the high bacterial inoculum used for the treatments is not representative of the situation of the NICU settings and additional investigations are required before extrapolating our results to real-life practice although the likely lower bacterial inoculum in real life conditions could be in favor of a higher bacteriophage activity and bacterial eradication. Finally, the long-term effect of bacteriophages in the NICU environment but also their effect on eukaryotic cells could be interesting points to explore.

To sum up, the present study constitutes a proof-of-concept for the use of bacteriophages on *S. capitis* NRCS-A environmental contamination. In the era of increasing resistance to antibiotics and disinfectants, the development of such innovative approach of decontamination using bacteriophages for biocontrol of specific pathogenic bacteria could find its full place in the landscape of infection prevention. Additional evaluations deserve to be performed to define the best and adequate use of biodecontamination and to evaluate its feasibility and efficacy in the NICU environment in conditions simulating real-life conditions.

## Data availability statement

The data presented in the study are deposited in the data.world repository, following the link https://data.world/mariechavignon/phage. The genomes of bacteriophages vB_ScaM-V1SC01, vB_ScaM-V1SC04 and vB_ScaM-V1SC05 are deposited in the GenBank repository, accession number OP323059, OP297178 and OP297179 respectively.

## Author contributions

MC and CK designed the study, analyzed the data and wrote the manuscript. MM, AT, FL, and MaB contributed to the design of the study. LB and TL contributed to the isolation, production and host range assessment of bacteriophages. MéB performed the bioinformatics analysis. MaB and FL reviewed the manuscript. All authors contributed to the article and approved the submitted version.

## Funding

This study was supported by the ANR (Agence Nationale de la Recherche) as part of the project NeoSCap [grant number ANR 19-CE17-0004-01] and the project PHAG-ONE [grant number ANR 20-PAMR-0009]. The authors thank the ANR (Agence Nationale de la Recherche) which supported this work as part of the project NeoSCap and the project PHAG-ONE and the Hospices Civils de Lyon Foundation which financed the PHAGEinLYON project.

## Conflict of interest

The authors declare that the research was conducted in the absence of any commercial or financial relationships that could be construed as a potential conflict of interest.

## Publisher’s note

All claims expressed in this article are solely those of the authors and do not necessarily represent those of their affiliated organizations, or those of the publisher, the editors and the reviewers. Any product that may be evaluated in this article, or claim that may be made by its manufacturer, is not guaranteed or endorsed by the publisher.

## Abbreviations

LRV, Log10 reduction values; MOI, Multiplicity of Infection; NICU, Neonatal Intensive Care Unit; SP, Surfanios Premium.
